# Impact of Fluid Overload on Mortality Among Critically Ill Pediatric Patients: An Observational Study at a Tertiary Care Hospital in Central India

**DOI:** 10.7759/cureus.82178

**Published:** 2025-04-13

**Authors:** Anitha Rajendran, Pratibha Bamne, Nitesh Upadhyay, Umesh Pandwar, Jyotsna Shrivastava

**Affiliations:** 1 Pediatrics, Gandhi Medical College, Bhopal, IND

**Keywords:** critical illness, cumulative fluid balance, fluid overload, length of stay, mortality, pediatric intensive care unit, prism iii score

## Abstract

Background

Fluid overload (FO) is a critical concern in pediatric intensive care units (PICUs), contributing to increased morbidity and mortality. Excessive fluid accumulation can exacerbate organ dysfunction, particularly affecting the cardiovascular, respiratory, and renal systems. While FO has been widely studied in adult populations, data on its burden, risk factors, and clinical outcomes in critically ill pediatric patients, particularly in low-resource settings like India, remain limited. This study aimed to assess the prevalence of significant cumulative FO percentage and its association with mortality, Pediatric Risk of Mortality (PRISM-III) score, and length of PICU stay.

Methods

This prospective observational study was conducted from June 2023 to October 2024 at the PICU of a tertiary care hospital in central India. A total of 230 children aged 1 month to 13 years who required intensive care were included. Demographic and clinical parameters, including fluid balance and PRISM-III scores, were recorded. FO was calculated based on cumulative fluid intake and output relative to baseline body weight. The association between FO and clinical outcomes was assessed using logistic regression analysis and receiver operating characteristic (ROC) curve analysis.

Results

The median (interquartile range (IQR)) cumulative FO at 24 hours, 48 hours, 7 days, and cumulative FO were 5.4% (3.4, 7.8), 5.3% (3.5-8), 5.7% (3.7-8.3), and 5.7% (3.7-8.4), respectively. The median PRISM-III score was 6 (IQR: 0-14). Among the 230 children, 13% died during follow-up. Non-survivors had significantly higher PRISM-III scores and FO percentages (p < 0.01). ROC analysis showed FO (area under the curve (AUC) = 0.72) and PRISM-III (AUC = 0.97) as strong mortality predictors. Multivariable regression identified 24 hours and overall cumulative FO and PRISM-III score as independent predictors of mortality.

Conclusion

Twenty-four-hour cumulative FO is a significant determinant of mortality in critically ill children, emphasizing the need for early monitoring and targeted management strategies in PICUs.

## Introduction

Fluid overload (FO) is a critical and prevalent issue in pediatric intensive care units (PICUs), with a well-documented impact on morbidity and mortality among critically ill children [1]. It occurs when the volume of administered fluids exceeds the body's capacity to manage, leading to adverse effects on vital organ systems such as the heart, lungs, and kidneys. In the PICU setting, where accurate fluid management is crucial, FO can exacerbate existing conditions and complicate recovery, contributing to longer hospital stays and higher mortality rates [1]. The pathophysiology of FO in critically ill children is multifaceted, often influenced by underlying diseases like sepsis, pneumonia, and acute respiratory distress syndrome (ARDS). These conditions disrupt the body's fluid balance due to increased vascular permeability, altered hemodynamics, and compromised renal function [2]. Furthermore, aggressive fluid resuscitation, a common intervention in critically ill patients, can lead to unintended fluid accumulation, especially in those with impaired fluid homeostasis. These pathophysiological changes necessitate precise fluid management to prevent the cascade of organ dysfunction triggered by FO.

Assessing and managing FO in the PICU presents significant challenges. Accurate assessment requires a combination of clinical evaluations, laboratory tests, and imaging studies to detect early signs of FO. This complexity often results in delayed recognition, which can lead to severe complications like pulmonary edema, cardiac dysfunction, and prolonged mechanical ventilation [3]. Bem and Lemson have shown that even a small increase in FO, such as exceeding 5% of the baseline body weight, can substantially elevate the risk of mortality and prolong ICU stays. These findings underscore the critical need for timely detection and intervention in fluid management [4].

Despite the growing recognition of FO’s impact, the clinical community lacks standardized guidelines for its management. There is considerable variability in practices related to fluid restriction, the timing of diuretic administration, and the use of renal replacement therapies [5]. This lack of consensus has contributed to inconsistent clinical outcomes, highlighting the need for evidence-based protocols tailored to the pediatric population. Effective management of FO requires balancing adequate tissue perfusion with the risks associated with excessive fluid administration, a challenge that demands individualized approaches based on the child's condition [6]. This study aims to estimate the prevalence of significant cumulative FO post-admission in critically ill pediatric patients and to investigate its association with key clinical outcomes, including mortality, Pediatric Risk of Mortality (PRISM) score, and length of stay in the PICU.

## Materials and methods

A consecutive sampling strategy was used, inviting all eligible children admitted to the PICU of Gandhi Medical College, Bhopal, India, during the study period (June 2023 to October 2024) to participate, ensuring that the sample was representative of the broader population of critically ill children in the hospital. Study tools included a combination of clinical assessments and diagnostic devices to capture relevant data. A pretested semi-structured questionnaire gathered demographic details, such as age, gender, and nutritional status, as well as clinical information, including underlying diagnoses. This questionnaire was pretested on 20 children, who were not part of the final analysis, to ensure clarity and reliability, with necessary adjustments made based on feedback.

Devices for diagnosing FO included meticulous fluid balance monitoring, where cumulative fluid intake (both oral and parenteral) and output were recorded through the hospital’s electronic medical record system. FO percentage was calculated at 24 hours, 48 hours, 7 days, and cumulatively using the following formula:

$\text{Fluid Overload (\%)} = \frac{\text{Cumulative Fluid Intake} - \text{Output}}{\text{Baseline Body Weight}} \times 100$

The PRISM III score, a validated tool for assessing illness severity, was used to evaluate each child upon admission. Diagnostic imaging, including chest X-rays and ultrasound, was performed as needed to detect FO signs such as pulmonary edema. Data were collected daily from admission throughout the stay via the hospital’s electronic records, capturing demographics, clinical parameters, fluid balance, PRISM-III scores, and outcomes (mortality, length of stay). Each participant’s data was anonymized with unique codes, stored in a secure, password-protected database accessible only to authorized personnel, with hard copies kept in locked storage. The study was approved by the Institutional Ethics Committee, Gandhi Medical College, Bhopal, and adhered to the Declaration of Helsinki. Informed consent was obtained from parents, with written assent for children aged 12-13, verbal assent for ages 7-12, and parental consent alone for children under seven.

Statistical analysis

Data analysis was performed using STATA version 14 (StataCorp, 2015. Stata Statistical Software: Release 14. College Station, TX: StataCorp LLC). Continuous variables were summarized as median and interquartile range (IQR) for non-normally distributed data. Categorical variables were presented as frequencies and percentages. The association between FO and clinical outcomes (mortality, PRISM score, length of stay) was assessed using the chi-square test and Wilcoxon rank-sum test. Bivariate and multivariate logistic regression were performed to identify independent predictors of mortality, adjusting for potential confounders such as age and gender. receiver operating characteristic (ROC) curve analysis was used to evaluate the discriminative ability of the PRISM score and overall cumulative FO percentage in predicting mortality. The area under the curve (AUC) was calculated to quantify the model's performance. All tests were two-tailed, with a p-value of less than 0.05 considered statistically significant.

## Results

A total of 250 children were approached, and finally, 230 children were included in the final analysis after applying the inclusion and exclusion criteria. The median (IQR) age was 2.5 (0.8, 7) years, and 57.4% were male children. The central nervous system, respiratory system, and sepsis were the three most commonly affected systems. The median (IQR) cumulative FO percentage at 24 hours, 48 hours, 7 days, and peak cumulative FO percentage were 5.4 (3.4, 7.8), 5.3 (3.5, 8), 5.7 (3.7, 8.3), and 5.7 (3.7, 8.4), respectively, and PRISM-III score was 6 (0-14). Among the 230 children, 13% died during the follow-up period. Table 1 shows the comparison of various socio-demographic characteristics of the study participants based on the outcome, i.e., survivors and the dead children at the end of follow-up. The PRISM-III score and 24-hour cumulative FO were significantly higher in non-survivors (p < 0.05), indicating a strong association with mortality. Other variables, such as age, gender, nutritional status, and system involvement, showed no statistically significant difference between survivors and non-survivors. The prevalence of significant cumulative FO (>10%) in our study was 16.1% (95% CI: 11.3% to 20.8%) (Table 1).

**Table 1:** Comparison of demographic, clinical, and physiological variables between survivors and non-survivors *chi-square test; ^Wilcoxan ranksum test IQR: interquartile range; PRISM: Pediatric Risk of Mortality; PICU: pediatric intensive care unit; CNS: central nervous system; GI: gastrointestinal

Variable	Survived (median (IQR)/n (%))	Dead (median (IQR)/n (%))	p-value*
Age in years	3 (0.8, 7)	2 (1, 4.5)	0.71^
Gender			
Male	111 (84.1)	21 (15.9)	0.13*
Female	89 (90.8)	9 (9.2)	
Nutritional status			
Undernourished	64 (88.9)	8 (11.1)	0.77*
Normal	135 (86)	22 (14)	
Obese	1 (100)	0	
PRISM score-III	6 (0, 14)	21 (14.5, 28.5)	<0.01^
Length of PICU stay (in days)	3 (2, 4)	3.5 (2, 6.5)	0.32^
24 hour cumulative fluid overload (%)	5.3 (3.3, 7.6)	7.6 (4.7, 8.8)	0.01^
48 hour cumulative fluid overload (%)	5.5 (3.5, 8.1)	4.8 (3.7, 8)	0.59^
7-day cumulative fluid overload (%)	6.1 (3.7, 8.4)	5.1 (3.7, 7.2)	0.52^
Overall cumulative fluid overload (%)	6.1 (3.7, 8.4)	4.1 (3.7, 7.2)	0.04^
Cumulative fluid overload % (<10%)	167 (86.5)	26 (13.5)	0.04*
Cumulative fluid overload % (>10%)	30 (81)	7 (19)	
Primary system involved			
Cardiovascular	15 (93.4%)	1 (6.3%)	0.90*
CNS	46 (83.6%)	9 (16.4%)	
Endocrine	5 (100%)	0	
GI and hepatic	20 (90.9%)	2 (9.1%)	
Hematology	18 (81.8%)	4 (18.2%)	
Infections	1 (100%)	0	
Poisoning	5 (83.3%)	1 (16.7%)	
Renal	6 (85.7%)	1 (14.3%)	
Respiratory	42 (84%)	8 (16%)	
Sepsis	42 (91.3%)	4 (8.7%)	

**Figure 1:**
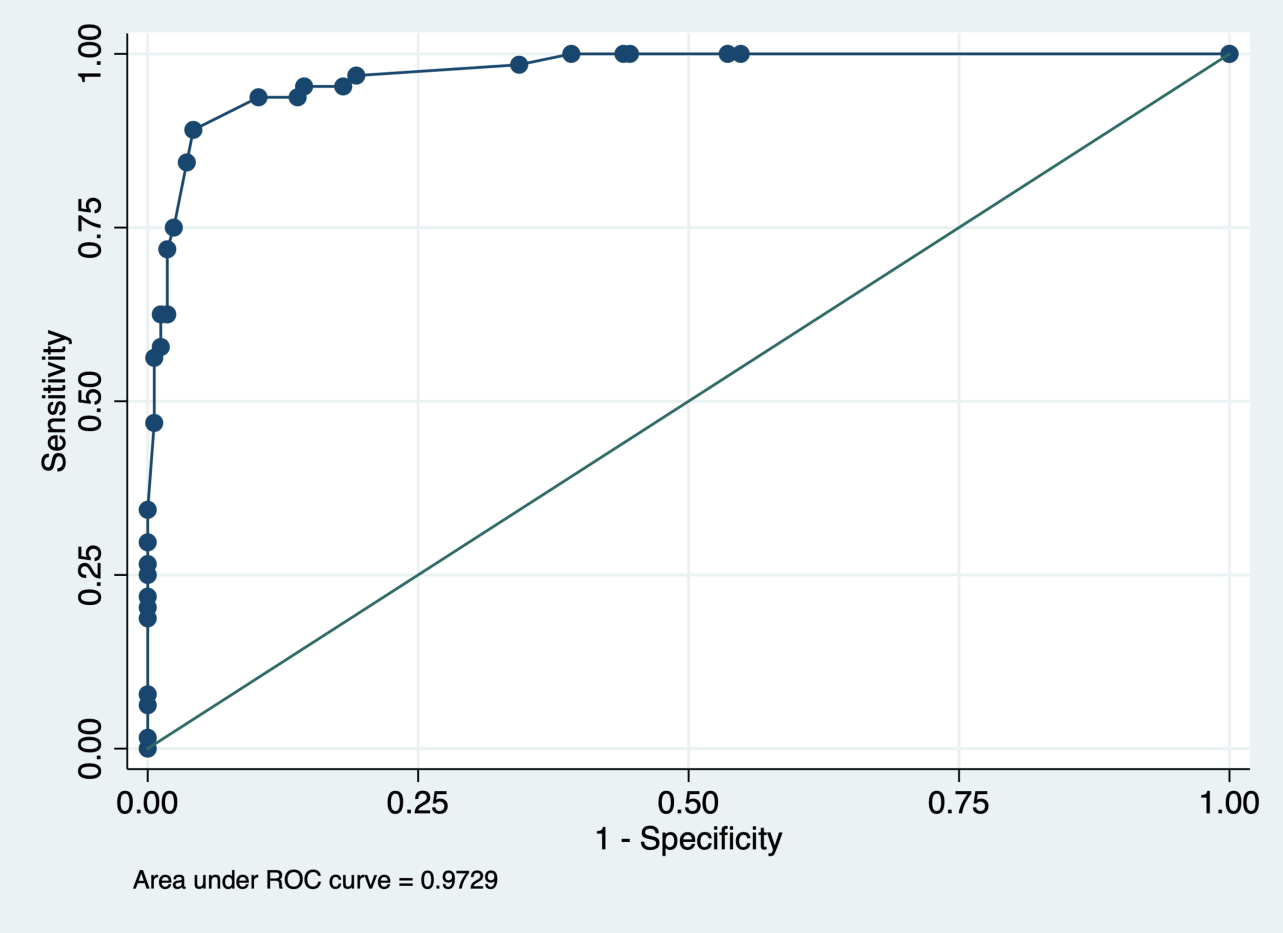
ROC curve of PRISM-III scores predicting the outcome ROC: receiver operating characteristic; PRISM: Pediatric Risk of Mortality

ROC analysis for predicting patient outcomes using the PRISM score showed an AUC of 0.97 (95% CI: 0.95-0.99), indicating excellent discriminative ability between survival and death. The optimal cut-off value for the PRISM score, determined by maximizing the Youden Index, was 13. At this threshold, the sensitivity was 89.1%, reflecting the percentage of correctly identified deaths, while the specificity was 95.8%, indicating the accuracy in identifying survivors. These results demonstrate that the PRISM score is a highly effective predictor of patient outcomes in this study population (Figure 1).

**Figure 2:**
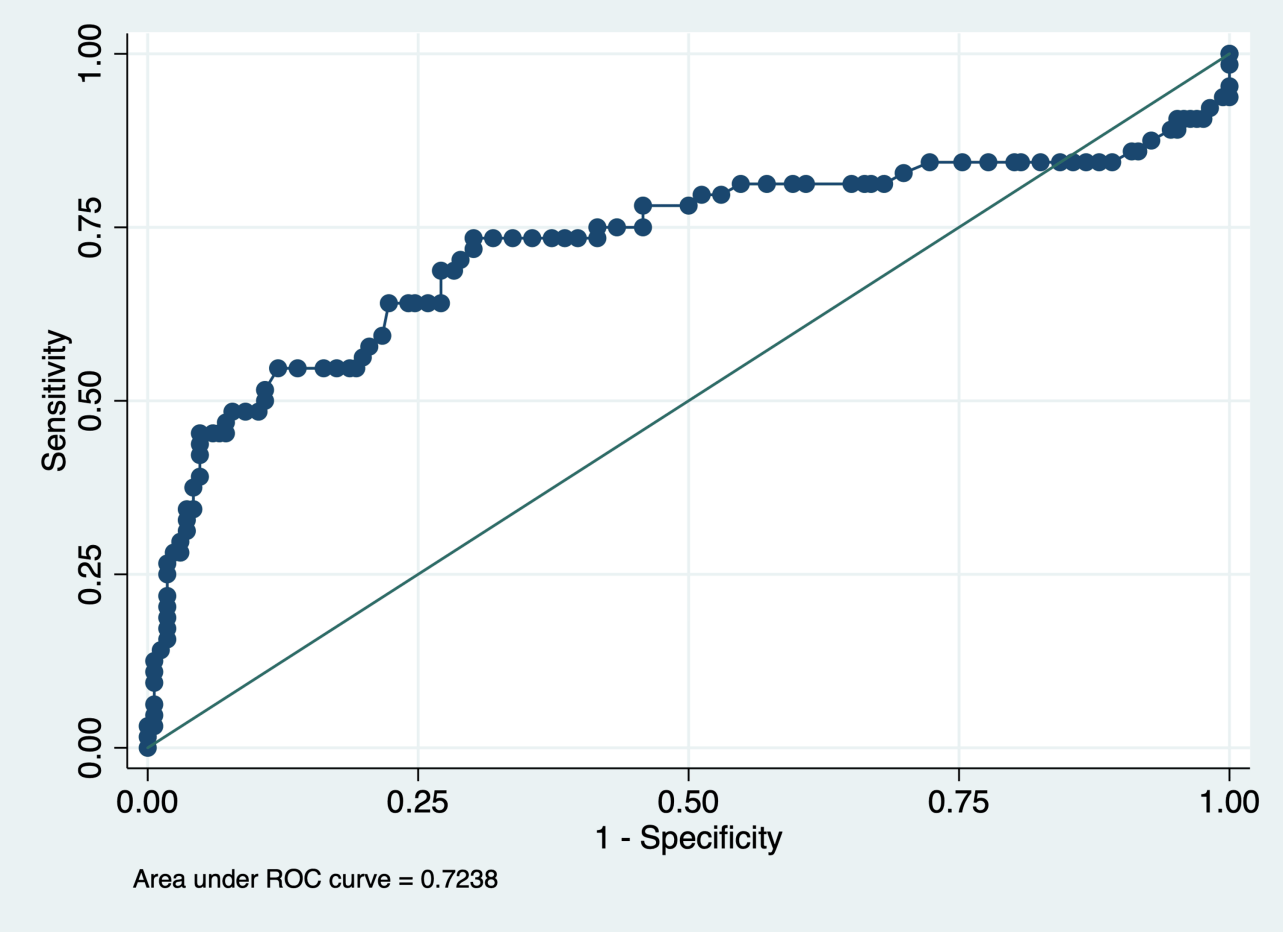
ROC curve of cumulative fluid overload percentage predicting the outcome ROC: receiver operating characteristic

ROC analysis for predicting patient outcomes using the overall cumulative FO percentage revealed an AUC of 0.72 (95% CI: 0.64-0.81), indicating a good ability to discriminate between survival and death. The optimal cut-off value for the overall cumulative FO percentage, determined by maximizing the Youden Index, was 7.0. At this threshold, the sensitivity was 68.8%, and the specificity was 72.9% (Figure 2).

The multivariable regression analysis (Table 2) identified overall cumulative FO and PRISM-III score as significant predictors of mortality in critically ill pediatric patients. In the bivariate regression analysis, an association between 24-hour, 48-hour, 7-day, and overall cumulative FO was found with mortality. In the multivariable model, 24-hour and cumulative FO were found to be statistically significant (AOR: 1.2, 95% CI: 1.1-3.2, p = 0.03, AOR: 2.32, 95% CI: 1.92-3.26, p = 0.02). The PRISM-III score was also independently associated with mortality (AOR: 1.6, 95% CI: 1.3-1.8, p < 0.01), reinforcing its predictive value. Other factors, including age, gender, BMI, and length of PICU stay, did not show a statistically significant independent association with mortality.

**Table 2:** Multivariable regression analysis to identify determinants of mortality (N = 230) BMI: body mass index; CI: confidence interval; PRISM: Pediatric Risk of Mortality; PICU: pediatric intensive care unit; IQR: interquartile range

Variable	Dead (n, %)	Crude odds ratio (95% CI)	p-value	Adjusted odds ratio (95% CI)	p-value
Age	2 (1, 4.5)	0.8 (0.9, 1.1)	0.98	1.1 (0.9, 1.3)	0.28
Gender					
Female	9 (9.2)	Reference		Reference	
Male	21 (15.9)	1.1 (0.6, 2.0)	0.71	0.5 (0.1, 1.6)	0.22
BMI					
Under-nourished	8 (11.1)	Reference		Reference	
Normal	22 (14)	0.8 (0.4, 1.4)	0.5	1.2 (0.3, 4.5)	0.79
Obese	0	-		-	
Fluid overload (%)					
24-hour cumulative fluid overload (%)	7.6 (4.7, 8.8)	1.1 (1.0, 1.2)	0.01	1.2 (1.1, 3.2)	0.03
48-hour cumulative fluid overload (%)	4.8 (3.7, 8)	1.7 (1.1, 2.5)	<0.01	1.0 (0.7, 1.4)	0.45
7-day fluid overload (%)	5.1 (3.7, 7.2))	1.2 (1.1, 1.3)	<0.01	1.1 (0.9, 1.2)	0.31
Overall cumulative fluid overload (%)	4.1 (3.7, 7.2)	2.2 (1.1, 1.3)	<0.01	2.32 (1.92, 3.26)	0.02
PRISM score-III	21 (14.5, 28.5)	1.5 (1.3, 1.7)	<0.01	1.6 (1.3, 1.8)	<0.01
Length of PICU stay	3.5 (2.4, 5)	1.1 (0.9, 2)	0.41	1.1 (1.1, 2.3)	0.04

## Discussion

This study assessed the degree of FO at seven days post-admission and evaluated its association with critical clinical outcomes, including mortality, PRISM score, and length of stay in a PICU within a tertiary care hospital in Central India. Our findings indicate a significant relationship between higher PRISM-III scores and longer PICU stays, as well as increased mortality risk. While FO at various time intervals was initially associated with mortality in crude analyses, this association was statistically significant only for 24-hour and cumulative FO in multivariable models after adjusting for confounding factors. Additionally, ROC analyses showed that PRISM-III had a high discriminatory ability for mortality prediction, while FO demonstrated a moderate predictive capacity. These results underscore the need for targeted, evidence-based management strategies for critically ill pediatric patients, especially concerning fluid management in high-risk PICU admissions.

Our study emphasizes FO as a critical factor influencing outcomes in critically ill pediatric patients, highlighting its association with increased mortality and prolonged PICU stays. Consistent with a growing body of literature, our findings underscore FO as a significant predictor of adverse outcomes in PICU settings. Studies by Chen et al. [7] and Lima et al. [8] demonstrate that FO, particularly beyond the initial 48 hours, correlates strongly with mortality in pediatric sepsis, suggesting that both the timing and progression of FO are crucial. Early FO destabilizes physiological functions, while later cumulative FO contributes to longer-term complications. Our study findings align with the study conducted by Sinitsky et al. [9], who observed that timely fluid management could mitigate adverse effects in patients with sepsis or renal impairment.

The impact of FO appears to vary by primary diagnosis and clinical characteristics. In cases of sepsis and acute kidney injury, compromised fluid clearance exacerbates FO's harmful effects, likely through mechanisms like inflammatory-mediated vascular leakage. Our findings align with Fernández-Sarmiento et al. [10] who noted heightened FO-related risks in children with renal impairment or sepsis, as these patients are particularly vulnerable to fluid accumulation. Although cumulative FO initially showed an association with mortality in our study, this association was not significant after adjusting for confounders, suggesting that FO’s impact may be influenced by factors such as illness severity, co-morbidities, and fluid management strategies, including diuretic use.

Mechanistically, the adverse effects of FO are primarily attributed to increased vascular permeability and endothelial dysfunction, particularly in the context of sepsis and critical illness. These alterations contribute to third-space fluid accumulation and interstitial edema, leading to organ dysfunction, most notably affecting the lungs, resulting in pulmonary edema, and the kidneys, causing impaired renal perfusion and acute kidney injury. Ahmad et al. [11] demonstrated a link between delayed FO and renal complications as well as extended PICU stays, emphasizing the need for early, targeted fluid management. Our findings reinforce this view, as cumulative FO’s association with prolonged PICU stay suggests that managing FO in its early stages may improve patient outcomes. Alobaidi et al. [12] similarly noted that delayed FO was linked to extended PICU stays and kidney complications, highlighting the importance of prompt intervention. However, the seven-day cumulative FO measurement in our study may have reduced sensitivity to the immediate effects seen in early FO, potentially explaining the weaker association with mortality.

While PRISM-III remains a reliable tool for mortality risk stratification, our study’s focus on FO underscores its practical value as a real-time, actionable metric in clinical practice. Strategies to reduce FO, such as early fluid restriction and cautious diuretic use, are essential, especially for patients with sepsis or renal impairment who are at higher risk for FO-related complications [13]. This study supports an increasing emphasis on personalized fluid management protocols in PICUs, particularly within the first 24 hours post-admission, to minimize FO accumulation and associated risks. Future research, especially multicenter studies across diverse pediatric populations, is necessary to further validate evidence-based guidelines for FO management and its role in improving PICU outcomes.

Strengths and limitations

This study has several strengths, including its prospective design, which allowed for a detailed temporal analysis of fluid accumulation and its outcomes. The inclusion of PRISM-III as an illness severity measure enhances the study's predictive scope, offering valuable insights into the independent and combined effects of FO at various intervals (24 hours, 48 hours, 7 days, and cumulative) and PRISM-III on mortality. The study’s setting in a tertiary care hospital serving a diverse pediatric population also strengthens its external validity, making the findings potentially generalizable to similar healthcare settings in resource-limited regions. However, there are some limitations. This study shows a lack of alternative calculations on percentages based on actual fluid requirement. The study adjusted for primary confounders such as age and gender; other potential confounders, including underlying comorbidities or additional interventions (e.g., diuretic use), were not considered, which may have influenced the findings. The single-center design may limit the generalizability of these results to other regions or healthcare settings with differing patient demographics or treatment protocols. Also, a few more studies on fluid restriction practice in the future are recommended.

## Conclusions

This study underscores FO as a critical factor influencing mortality and PICU outcomes in critically ill pediatric patients. Our findings highlight that FO, particularly in the initial several hours of admission, significantly affects outcomes in terms of mortality. This is more applicable to children with sepsis or renal impairment. Our study emphasizes that FO monitoring and management as part of PICU care are crucial in reducing FO-related complications. A proactive approach is needed for individualized fluid management, prioritizing early intervention to prevent adverse outcomes linked to FO. Further multicenter studies with larger sample sizes are essential to validate these findings and establish evidence-based, standardized protocols for FO management in pediatric critical care settings.

## References

[1] Charaya S, Angurana SK (2024). Fluid overload in critically ill children: a narrative review. J Pediatr Crit Care.

[2] Gomes RA, Azevedo LF, Simões BP (2023). Fluid overload: clinical outcomes in pediatric intensive care unit. J Pediatr (Rio J).

[3] Arikan AA, Zappitelli M, Goldstein SL, Naipaul A, Jefferson LS, Loftis LL (2012). Fluid overload is associated with impaired oxygenation and morbidity in critically ill children. Pediatr Crit Care Med.

[4] Bem RA, Lemson J (2024). Evaluating fluid overload in critically ill children. Curr Opin Pediatr.

[5] Claure-Del Granado R, Mehta RL (2016). Fluid overload in the ICU: evaluation and management. BMC Nephrol.

[6] Lehr AR, Rached-d'Astous S, Barrowman N (2022). Balanced versus unbalanced fluid in critically ill children: systematic review and meta-analysis. Pediatr Crit Care Med.

[7] Chen J, Li X, Bai Z (2016). Association of fluid accumulation with clinical outcomes in critically ill children with severe sepsis. PLoS One.

[8] Lima L, Menon S, Goldstein SL, Basu RK (2021). Timing of fluid overload and association with patient outcome. Pediatr Crit Care Med.

[9] Sinitsky L, Walls D, Nadel S, Inwald DP (2015). Fluid overload at 48 hours is associated with respiratory morbidity but not mortality in a general PICU: retrospective cohort study. Pediatr Crit Care Med.

[10] Fernández-Sarmiento J, Sierra-Zuñiga MF, Salazar González MP, Lucena N, Soares Lanziotti V, Agudelo S. (2023). Association between fluid overload and mortality in children with sepsis: a systematic review and meta-analysis. BMJ Paediatr Open.

[11] Ahmad A, Gable B, Bost J, Tuchman S, Klugman D (2019). 1467: antibiotic-associated acute kidney injury in critically ill children with cardiac disease. Crit Care Med.

[12] Alobaidi R, Basu RK, DeCaen A, Joffe AR, Lequier L, Pannu N, Bagshaw SM (2020). Fluid accumulation in critically ill children. Crit Care Med.

[13] Wong JJ, Ho SX, Lee AOC (2019). Positive fluid balance is associated with poor clinical outcomes in paediatric severe sepsis and septic shock. Ann Acad Med Singapore.

